# Supplementary material for: Harnessing Paenarthrobacter ureafaciens YL1 and Pseudomonas koreensis YL2 Interactions to Improve Degradation of Sulfamethoxazole
Source: Microorganisms. 2022 Mar 18;10(3):648. doi: 10.3390/microorganisms10030648 (PMC8953276; doi:10.3390/microorganisms10030648)
Supplement: Supplementary file 1 [file microorganisms-10-00648-s001.zip › microorganisms-1640284-supplementary.pdf]

Supplementary material

-Journal name: ***Microorganisms***

-**Manuscript Title: Harnessing *Paenarthrobacter ureafacien*s YL1 and *Pseudomonas koreensis* YL2 interactions to improve degradation of sulfamethoxazole**

-The name(s) of the author(s): Lan Yu[1], Yingning Wang[1], Xiaoqing Shan[1], Fang Ma[1,*], Haijuan Guo[2,*]

-The affiliation(s) and address(es) of the author(s):

[1] State Key Laboratory of Urban Water Resource and Environment, School of Environment, Harbin Institute of Technology, Harbin 150090, China

[2] College of Energy and Environmental Engineering, Hebei University of Engineering, Handan 056038, China

-The e-mail address of the corresponding author

[*]Corresponding author. E-mail: mafang@hit.edu.cn; guohaijuan@163.com

Table S1 The CARD analysis53 of the strain YL2: the Resistance Gene Identifier (RGI) for perfect and strict hits to known resistance genes.

RGI Criteria	ARO Term	Detection Criteria	AMR Gene Family	Drug class	Resistance Mechanism	% Identity of Matching Region	% Length of Reference Sequence
Strict	adeF	protein homolog model	resistance-nodulation-cell division (RND) antibiotic efflux pump	fluoroquinolone antibiotic, tetracycline antibiotic	antibiotic efflux	66.6	100.28
Strict	adeF	protein homolog model	resistance-nodulation-cell division (RND) antibiotic efflux pump	fluoroquinolone antibiotic, tetracycline antibiotic	antibiotic efflux	43.1	97.45
Strict	FosA	protein homolog model	fosfomycin thiol transferase	fosfomycin	antibiotic inactivation	69.12	102.22
Strict	Pseudomonas aeruginosa soxR	protein homolog model	ATP-binding cassette (ABC) antibiotic efflux pump, major facilitator superfamily (MFS) antibiotic efflux pump, resistance-nodulation-cell division (RND) antibiotic efflux pump	fluoroquinolone antibiotic, cephalosporin, glycylcycline, penam, tetracycline antibiotic, acridine dye, rifamycin antibiotic, phenicol antibiotic, triclosan, disinfecting agents and intercalating dyes	antibiotic target alteration, antibiotic efflux	68.79	95.51
Strict	Acinetobacter baumannii AbaQ	protein homolog model	major facilitator superfamily (MFS) antibiotic efflux pump	fluoroquinolone antibiotic	antibiotic efflux	72.81	101.38
